# Physio- und sporttherapeutische Interventionen zur Behandlung eines Karpaltunnelsyndroms: Eine systematische Übersichtsarbeit

**DOI:** 10.1007/s00482-022-00637-x

**Published:** 2022-03-14

**Authors:** Julia Katharina Gräf, Kerstin Lüdtke, Bettina Wollesen

**Affiliations:** 1 Institut für Bewegungswissenschaft, Arbeitsbereich Bewegungs- und Trainingswissenschaft, Universität Hamburg, Hamburg, Deutschland; 2 Institut für Gesundheitswissenschaften, Fachbereich Physiotherapie, Universität zu Lübeck, Lübeck, Deutschland

**Keywords:** Konservative Behandlung, Verbesserung der Funktionalität/Hand, Symptomkontrolle, Manuelle Therapie, Yoga, Conservative treatment, Recovery of function/hand, Symptom control, Manual therapies, Yoga

## Abstract

**Hintergrund:**

Die Behandlung des Karpaltunnelsyndroms (KTS) besteht in der Regel in einer operativen Dekompression des Nervs oder Schienung und einer zusätzlichen medikamentösen Therapie. Physio- und Sporttherapie können eine nichtinvasive und gleichzeitig nebenwirkungsarme Alternative darstellen.

**Ziel:**

Die vorliegende Übersichtsarbeit fasst aktuelle Studien zur Wirksamkeit von physio- und sporttherapeutischen Interventionen für die Therapie des KTS systematisch zusammen und fokussiert auf die Reduktion der Symptome sowie als sekundäres Outcome auf die Verbesserung der Funktionalität der Hand.

**Material und Methoden:**

Das systematische Review integriert randomisierte, kontrollierte Studien mit physio- oder sporttherapeutischen Interventionen, die in den elektronischen Datenbanken PubMed, CINAHL und Web of Science bis Februar 2021 publiziert wurden. Den Richtlinien von Preferred Reporting Items for Systematic Reviews and Meta-Analyses (PRISMA) und der Cochrane Collaboration folgend wurden eine systematische Suche der Literatur, eine Datenextraktion und eine Bewertung des „risk of bias“ anhand des Cochrane Risk of Bias Tool von zwei unabhängigen Reviewern durchgeführt.

**Ergebnisse:**

Von 461 identifizierten Studien konnten *n* = 26 in die qualitative Analyse einbezogen werden. Das Biasrisiko über die einzelnen Studien ist als moderat bis gering einzustufen. Verzerrungspotenzial ergibt sich teilweise durch eine unzureichende Verblindung der Patient:innen und des Studienpersonals sowie durch eine selektive Berichterstattung der Studienergebnisse und der Durchführung. Die manuelle Therapie erwies sich im Vergleich zu einem operativen Eingriff als schneller und langfristig gleichermaßen zielführend in Bezug auf Schmerzreduktion und Funktionsverbesserung. Auch Mobilisationstechniken, Massagetechniken und das Kinesiotaping sowie Yoga als therapeutische Interventionen zeigten positive Effekte.

**Schlussfolgerung:**

In der Therapie eines leichten bis mittelschweren KTS zeichnen sich physio- und sporttherapeutische Interventionen vor allem durch Erfolge bereits nach 2‑wöchiger Behandlung aus, zudem durch vergleichbare Erfolge wie nach operativem Eingriff und 3‑monatiger postoperativer Behandlung. Zudem sind Patient:innen keinen Operationsrisiken ausgesetzt. Das Review ist im International Prospective Register of Systematic Reviews (PROSPERO) mit der Nr. 42017073839 registriert.

**Zusatzmaterial online:**

Die Online-Version dieses Beitrags (10.1007/s00482-022-00637-x) enthält die Datenextraktionstabelle.

## Abstract (en)

**Background:**

The treatment of carpal tunnel syndrome (CTS) usually involves surgical decompression of the nerve or splinting and additional medication. Physiotherapy and sports therapy could be non-invasive and alternative treatment approaches with a simultaneous low risk of side effects.

**Objective:**

The review systematically summarizes the current studies on the effectiveness of physiotherapy and sports therapeutic interventions for treatment of CTS and focuses on the reduction of symptoms and, as a secondary outcome, improvement of hand function.

**Material and methods:**

The systematic review includes randomized controlled trials reporting on physiotherapy or sports therapy interventions published prior to February 2021 in the electronic databases PubMed, CINAHL and Web of Science. Following the guidelines of preferred reporting items for systematic reviews and meta-analyses (PRISMA) and the Cochrane Collaboration, a systematic search of the literature, data extraction and evaluation of the risk of bias using the Cochrane risk of bias tool were conducted by two independent researchers.

**Results:**

Out of 461 identified studies 26 were included in the qualitative analysis. The risk of bias in the individual studies was graded as moderate to low. Potential bias might arise due to inadequate blinding of patients and study personnel in some cases as well as due to selective reporting of study results and procedures. Manual therapy proved to be faster and equally effective in reducing pain and improving function in the long term compared to surgery. Mobilization techniques, massage techniques, kinesiotaping and yoga as therapeutic interventions also showed positive effects on symptoms.

**Conclusion:**

For the management of mild to moderate CTS, physiotherapy and sports therapeutic interventions are characterized primarily by success after as little as 2 weeks of treatment as well as comparable success to surgery and 3 months of postoperative treatment. In addition, patients are not exposed to surgical risks. The protocol was registered in the International Prospective Register of Systematic Reviews (PROSPERO) with the number 42017073839.

Das Karpaltunnelsyndrom (KTS) ist eine der häufigsten und kostenintensivsten pathophysiologischen Mononeuropathien. Sie betrifft jeden sechsten Erwachsenen im arbeitsfähigen Alter [6, 7, 13, 22]. Ein operativer Eingriff stellt die derzeit gängigste Behandlungsmethode dar. Jedoch sind viele Behandelte auch postoperativ bis zu 2 Monate arbeitsunfähig [16]. Physiotherapie wird zwar klinisch oft eingesetzt, die bestehende Evidenz ist jedoch widersprüchlich und es gibt keine aktuelle und systematische Übersicht über die Effektivität einzelner Behandlungsformen.

## Theoretischer Hintergrund und Fragestellung

Bei einem KTS wird der N. medianus im Bereich des Karpaltunnels aufgrund eines Missverhältnisses zwischen Weite und Inhalt des Tunnels komprimiert. Typische Frühsymptome sind nächtliche schmerzhafte Missempfindungen (Brachialgia paraesthetica nocturna), die im weiteren Verlauf auch tagsüber persistieren können. Schließlich kommt es zu zunehmenden sensorischen und motorischen Ausfallserscheinungen in Bereichen des Daumens bis zum Mittelfinger und in Teilen des Ringfingers [5].

Viele etablierte physio- und sporttherapeutische Interventionen werden bereits in der Praxis eingesetzt

Vorliegende Übersichtsarbeiten zum KTS beinhalten hauptsächlich Studien zur operativen Nachversorgung [41], vergleichen nichtinterventionelle Studien, die auch Medikamente oder Injektionen beinhalten [25], und kombinieren Interventionen, die eine Beurteilung spezifischer Wirkmechanismen einzelner Therapien nicht zulassen. Weitere Kritikpunkte sind eine geringe Anzahl eingeschlossener Studien [38] oder mangelnde Aktualität, weil neuere Studien noch nicht berücksichtigt wurden [32, 36, 37, 40]. Die ebenfalls nicht mehr aktuelle S3-Leitlinie „Karpaltunnelsyndrom“ von 2012 berichtet noch zurückhaltend über physio- oder sporttherapeutische Behandlungsansätze und spricht von einer Überlegenheit operativer Methoden [9], obwohl in der Praxis bereits viele etablierte physio- und sporttherapeutische Interventionen eingesetzt werden, deren Effektivität in Interventionsstudien belegt werden konnte. Zu nennen sind insbesondere manuelle Therapieformen, wie Nerven-Sehnen-Gleitübungen [11], Mobilisationen (im Verlauf des Nervs, Karpalknochenmobilisationen, Weichteilmobilisationen; [40]), aber auch physikalische Therapien, wie Laser- und Ultraschalltherapie [12, 14, 31, 40, 43], oder sporttherapeutische Behandlungen, wie Yoga. Darüber hinaus scheint Kinesiotaping eine Behandlungsoption für das KTS zu sein [1, 21, 27, 29, 50, 59].

Das Ziel dieser systematischen Übersichtsarbeit ist daher, einen aktuellen Überblick über randomisierte klinische Studien zur Effektivität physio- oder sporttherapeutischer Interventionen im Vergleich zu anderen Therapieformen, Placebo oder einer Operation zu geben. Zielparameter zur Beurteilung der klinischen Effektivität sind Schmerzminderung und Handfunktion.

## Studiendesign und Untersuchungsmethoden

Die systematische Literaturübersicht folgte den Vorgaben des Cochrane-Handbuchs [24] und wurde nach den Empfehlungen des Preferred-Reporting-Items-for-Systematic-Reviews-and-Meta-Analyses(PRISMA)-Statements berichtet [34]. Das Protokoll wurde vor Beginn der Datenbankrecherche im International Prospective Register of Systematic Reviews (PROSPERO) registriert (42017073839).

### Ein- und Ausschlusskriterien

Eingeschlossen wurden deutsch- oder englischsprachige randomisierte, kontrollierte Studien, die eine physiotherapeutische oder sporttherapeutische Intervention mit einer anderen nichtoperativen Intervention, einer anderen physio- oder sporttherapeutischen Therapieform, Placebo/Warteliste oder einer Operation verglichen.

Interventionen, die primär in Kombination mit Operationen, Medikation oder Schienenversorgung durchgeführt wurden, wurden ausgeschlossen, da die Wirksamkeit der einzelnen Therapien nicht mehr erkennbar war.

Patient:innen mussten eine klinische oder physiologische Diagnose eines KTS (anhand einer etablierten Diagnosemethode wie des Tinel-Zeichens oder elektrophysiologischer Tests) aufweisen und durften zudem keine zusätzlichen klinisch relevanten internistischen und/oder muskuloskeletalen Erkrankungen haben. Eine vorherige Operation galt nicht als Ausschlusskriterium. Ausgeschlossen wurden Studien mit Kindern, Schwangeren oder einem KTS als Sekundärerkrankung.

Als Endpunkt musste in den Studien zumindest ein Wert für die Schmerzintensität oder die Handfunktion beschrieben sein.

### Informationsquellen

Die Studien wurden ohne Datumsbeschränkung anhand einer elektronischen Suche mit den oben genannten Ein- und Ausschlusskriterien in den Datenbanken PubMed, CINAHL und Web of Science sowie anhand einer Handsuche durch zwei Personen unabhängig identifiziert. Einbezogen wurden bis zum 24. Januar 2021 veröffentlichte Publikationen (vgl. Online-Zusatzmaterial). Die Handsuche umfasste das Screening der Literaturlisten eingeschlossener Studien und die Suche in den Inhaltsverzeichnissen aller Zeitschriften, in denen die eingeschlossenen Studien veröffentlicht worden waren.

**Figure 1:**
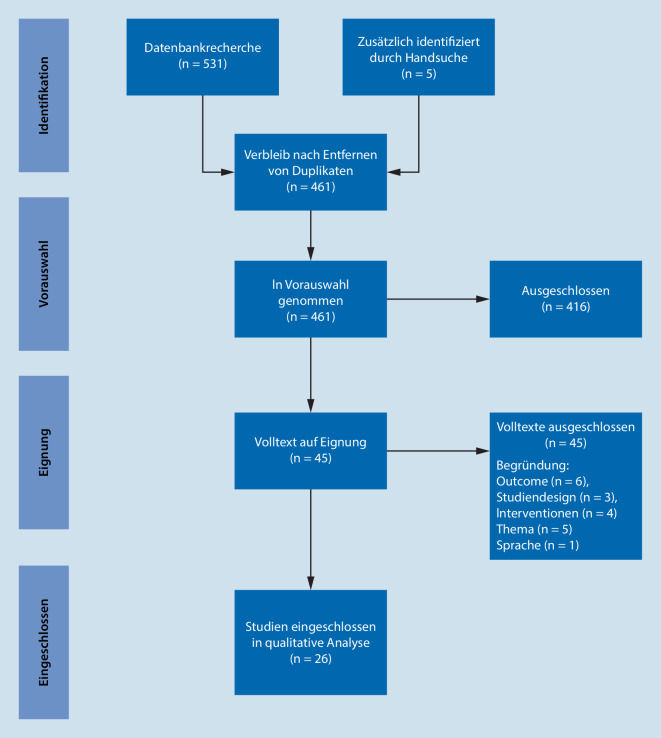

Die Auswahl der Studien erfolgte durch zwei unabhängige Reviewer zunächst nach Titel und Abstract. Anschließend wurden die Volltexte der ausgewählten Studien anhand der Ein- und Ausschlusskriterien überprüft (Abb. 1). Unstimmigkeiten wurden in einem Konsensmeeting ausgeräumt.

### Datenextraktion

Anhand einer Datenextraktionstabelle extrahierten zwei Personen unabhängig die folgenden Informationen:

- Allgemeine Informationen (Autor:innen, Jahr der Veröffentlichung, Journal)
- Beschreibung der Personenstichproben (Interventionsgruppe, Kontrollgruppe, diagnostische Kriterien)
- Beschreibung der Intervention (Intervention, Kontrollintervention, Interventionsdauer, Nachuntersuchungszeitraum)
- Messinstrumente (VAS, BCTQ SSS, BCTQ FSS, NPRS, DN4)
- Ergebnisse Schmerz/Funktion (Mittelwerte, Standardabweichungen, Konfidenzintervalle und Signifikanz)

### Bewertung der methodischen Studienqualität

Die Beurteilung der methodischen Qualität eingeschlossener Studien erfolgte ebenfalls durch zwei unabhängige Personen anhand des Cochrane-Instruments zur Bewertung des Biasrisikos [23]. Unstimmigkeiten wurden im Konsens ausgeräumt. Die Übereinstimmung der Reviewer:innen vor Konsensfindung wurde anhand von Kappa-Werten [4, 30] berechnet.

### Synthese der Ergebnisse

Die Ergebnissynthese erfolgte in strukturierter narrativer Form. Dafür wurden unter Berücksichtigung der methodischen Qualität der einzelnen Studien der primäre Zielparameter (Schmerzintensität) und der sekundäre Parameter (Funktionalität der Hand) mithilfe der eingesetzten Bewertungsskalen (Schmerzskalen und Funktionalitätsskalen; siehe Messinstrumente) verglichen. Die klinische Relevanz wurde auf Basis der Veränderungen in den subjektiven Skalen eingestuft [28, 45, 48]. Hierzu wurden zuvor publizierte Werte für die Schmerzintensität (VAS; [48]) herangezogen; Differenzen zwischen Vor- und Nachuntersuchung von 0,5 Punkten bei Ausgangswerten zwischen 0 und 4 Punkten sowie Differenzen von 2 Punkten bei Ausgangswerten über 4 Punkte wurden als klinisch relevant eingestuft. Weiterhin galten Differenzen in der Schmerzintensität (NPRS; [45]) von einem Punkt zwischen Prä- und Postuntersuchung als klinisch relevant. Für die Symptomstärke (BCTQ SSS; [28]) galten Differenzen von 0,46 × Ausgangswert als Richtwert für die minimale klinisch relevante Differenz zwischen Ausgangs- und Endwert. Die zugrunde gelegte Einstufung der klinischen Relevanz bezüglich der Funktionalität der Hand (BCTQ FSS; [28]) basierte auf der minimalen Differenz der Ausgangs- und Endwerte, ermittelt durch 0,28 × Ausgangswert.

## Ergebnisse

### Auswahl der Studien

Die Recherche identifizierte nach Entfernung von Duplikaten insgesamt 461 Publikationen. Hier erfüllten 26 Studien nach abgeschlossenem Volltextscreening die Einschlusskriterien zur weiteren Ergebnissynthese (Abb. 1).

### Verzerrungsrisiko innerhalb der Studien

Die methodische Qualität der eingeschlossenen Studien für die einzelnen Dimensionen war heterogen, mit überwiegend moderatem bis geringem Biasrisiko (Tab. 1). Ein unklares Biasrisiko wurde bei vielen der eingeschlossenen Studien für die Dimension der selektiven Berichterstattung festgestellt, da kein Studienprotokoll verfügbar war. Ebenso unklar war in einigen Berichten die Angabe zur verdeckten Gruppenzuteilung, welche die Reviewer als hohes Verzerrungsrisiko einstuften. Bei Studien zu physio- und sporttherapeutischen Interventionen sowie zu operativen Verfahren ist eine Verblindung der Patient:innen und des Studienpersonals meist nicht möglich. Daraufhin könnten Verzerrungen in der subjektiven Bewertung der Patient:innen durch Erwartungen und erfüllte/nichterfüllte Vorlieben entstehen. Dennoch wurde dies als geringes Biasrisiko gewertet, da in solchen therapeutischen Studien kaum Möglichkeiten zur Verblindung von Patient:innen und Behandler:innen bestehen. Die Übereinstimmung zwischen den beiden Bewertern für die Einschätzung des Biasrisikos war hoch mit Cohens Kappa von 0,80 [4, 30].

**Table 1:** 

Autor:innen, Jahr, Land	1	2	3	4	5	6	7	Kommentare
Aktürk et al., 2018, Türkei [2]	**+**	*?*	**+**	**+**	**+**	*?*	–	–
Alam et al., 2018, Pakistan [3]	**+**	*?*	**+**	*?*	**+**	*?*	–	–
Atya et al., 2011, Ägypten [8]	**+**	*?*	**+**	*?*	**+**	*?*	**+**	–
Bardak et al., 2009, Türkei [10]	**+**	**+**	**+**	**+**	**+**	*?*	**+**	–
Burke et al., 2007, USA [15]	**+**	*?*	**+**	**+**	**+**	*?*	**+**	–
Fernández-de-las-Peñas et al., 2015, Spanien [17]	**+**	**+**	**+**	**+**	**+**	–	**+**	–
Fernández-de-las-Peñas et al., 2017, Spanien [18]	**+**	**+**	**+**	**+**	**+**	**+**	**+**	–
Fernández-de-las-Peñas et al., 2017, Spanien [19]	**+**	**+**	**+**	**+**	**+**	**+**	**+**	–
Garfinkel et al., 1998, USA [20]	**+**	**+**	**+**	**+**	–	*?*	**+**	–
Külcü et al., 2016, Türkei [29]	**+**	**+**	**+**	**+**	**+**	*?*	**+**	–
Jiménez del Barrio et al., 2018, Spanien [26]	**+**	**+**	**+**	**+**	–	**+**	**+**	–
Mohamed et al., 2016, Ägypten [33]	*?*	*?*	*?*	*?*	*?*	**+**	**+**	–
Moraska et al., 2008, USA [35]	*?*	*?*	*?*	–	**+**	*?*	**+**	Verblindung Teilnehmer ≠ Personal
Pratelli et al., 2015, Italien [42]	–	*?*	**+**	**+**	**+**	*?*	**+**	–
Sakr et al., 2019, Ägypten [44]	*?*	*?*	*?*	*?*	**+**	*?*	**+**	–
Schmid et al., 2012, Australien [46]	**+**	**+**	**+**	**+**	**+**	**+**	**+**	–
Shem et al., 2020, USA [49]	**+**	**+**	**+**	**+**	*?*	**+**	**+**	–
Tal-Akabi et al., 2000, Schweiz [51]	**+**	*?*	–	**+**	**+**	*?*	**+**	–
Talebi et al., 2020, Iran [52]	**+**	**+**	**+**	**+**	**+**	**+**	**+**	–
Vikranth et al., 2015, Indien [53]	**+**	**+**	–	*?*	**+**	*?*	**+**	–
Wolny et al., 2014, Polen [58]	**+**	**+**	*?*	**+**	*?*	*?*	**+**	–
Wolny et al., 2017, Polen [57]	**+**	**+**	**+**	**+**	**+**	**+**	**+**	–
Wolny et al., 2018, Polen [54]	**+**	**+**	**+**	**+**	**+**	**+**	**+**	–
Wolny et al., 2018, Polen [55]	**+**	**+**	**+**	**+**	**+**	**+**	–	–
Wolny et al., 2019, Polen [56]	**+**	**+**	–	**+**	**+**	**+**	–	–
Yildirim et al., 2018, Türkei [59]	*?*	*?*	–	**+**	*?*	*?*	**+**	–

*+* geringes Verzerrungspotenzial, *–* hohes Verzerrungspotenzial, *?* unklares Verzerrungspotenzial, *1* Generierung der Randomisierungssequenz, *2* verdeckte Gruppenzuteilung, *3* Verblindung von Studienpersonal/-teilnehmer:innen, *4* Verblindung der Endpunkterhebung/-bewertung, *5* fehlende Daten bei der Endpunkterhebung/-bewertung, *6* selektives Berichten von Endpunkten, *7* andere Ursachen für Bias

### Studieninhalte

Die meisten Studien untersuchten eine manuelle Therapieform (*n* = 18). Davon wendeten *n* = 10 Studien neurodynamische Techniken an, *n* = 3 Studien eine Kombination von Neurodynamik und Kinesiotaping und *n* = 5 Studien manuelle Mobilisationstechniken, wie Weichteilmobilisationen entlang des N. medianus (Schulter, Ellenbogen, Unterarm, Handgelenk oder Finger), Karpalknochenmobilisationen oder Mobilisationen assoziierter anatomischer Bereiche. Insgesamt 5 Studien untersuchten eine Form der physikalischen Therapie (Ultraschall- und/oder Lasertherapie) und nur eine Studie evaluierte den Effekt einer sporttherapeutischen Intervention (Yoga). Insgesamt ließ sich eine deutliche Heterogenität der Messinstrumente (VAS, BCTQ SSS, BCTQ FSS, NPRS, DN4, PRS, FBS, PSFS, STP) sowie der Gruppengrößen feststellen, weshalb an dieser Stelle keine Metaanalyse der Daten möglich und sinnvoll erschien.

### Manuelle Therapieformen

#### Neurodynamik

Insgesamt 8 Studien untersuchten den Effekt des Einsatzes der Neurodynamik, die entweder als Selbstmobilisation von den Patient:innen oder von Therapeut:innen durchgeführt wurde. Die Intervention wurde verglichen mit dem Tragen einer Schiene [10, 46], der Behandlung durch eine Scheintherapie oder keiner Therapie [33, 49, 51, 54–56]. Die Interventionszeiträume variierten von einer [46] über 3 [51] und 6 [10, 33, 49] bis zu 10 Wochen [54–56] mit insgesamt 658 Patient:innen. Weitere 3 Studien verglichen die neurodynamische Mobilisation mit der Karpalknochenmobilisation [44, 53] oder der Weichteilmobilisation [52] mit insgesamt 90 Patient:innen über einen Zeitraum von 2 [53] und 4 Wochen [44, 52].

Insgesamt ließen sich durch neurodynamische Techniken bereits nach 2 Wochen klinisch relevante, positive Veränderungen in der Linderung der Symptomstärke (BCTQ SSS), Schmerzen, Hypästhesien und Missempfindungen erreichen [46, 52, 55, 56]. Zusätzlich zeigte die spezifische Bewertung der Schmerzen via VAS und NPRS klinisch relevante Verbesserungen durch den Einsatz neurodynamischer Techniken [44, 51–54, 56]. Weiterhin konnte eine Verbesserung der Funktionalität der Hände (BCTQ FSS) gegenüber der Standardversorgung, einer Schienung des Handgelenks oder keiner Behandlung in mehreren Studien mit einem geringen Verzerrungspotenzial nachgewiesen und als klinisch relevant eingestuft werden [10, 52, 54, 56, 59]. Zu berücksichtigen sind zudem die hochsignifikanten Ergebnisse von Mohammed et al. [33] hinsichtlich der verringerten Schmerzintensität und Symptomstärke, die jedoch aufgrund des erheblichen Verzerrungspotenzials als weniger aussagekräftig zu werten sind.

Daneben erwies sich die Karpalknochenmobilisation [44, 51, 53] als klinisch relevant für die Reduktion der Schmerzintensität [44, 51, 53]. Jedoch ist darauf hinzuweisen, dass die Studien [44, 51, 53] ein erhebliches Verzerrungspotenzial mit sehr vielen Unklarheiten bezüglich der methodischen Vorgehensweise aufweisen. Vor diesem Hintergrund sind diese Ergebnisse in ihrer Aussagekraft herabzustufen.

#### Neurodynamik und Kinesiotaping

Weitere 3 Studien [2, 29, 59] untersuchten die Wirkung einer Kombination aus Neurodynamikübungen und Kinesiotaping bei insgesamt 105 Patient:innen über einen Interventionszeitraum von 4 [29], 5 [2] und 6 Wochen [59]. Die Studienergebnisse von Külcü et al. [29] zeigen eine klinische Relevanz sowohl in Bezug auf die Verringerung der Schmerzintensität als auch bezüglich der Verbesserung der Funktionalität nach einer kombinierten Anwendung von Neurodynamik und Kinesiotaping. Die Studie weist ein geringes Verzerrungspotenzial auf. Zwei weitere Untersuchungen bestätigten eine klinische Relevanz in der Verbesserung der Funktionalität [2, 59]. Mit der Kombination beider Interventionen wurden nach einer Mindestbehandlungsdauer von 4 Wochen insgesamt größere Reduktionen der Symptomstärke und Verbesserungen der Funktionalität erzielt als mit alleiniger Neurodynamik oder Schienung.

#### Mobilisation

Drei Studien befassten sich mit dem Effekt einer 3‑wöchigen manuellen Therapie, bei der entweder Weichteilmobilisationen oder Mobilisationen von Gelenken entlang des N. medianus (Schulter, Ellenbogen, Unterarm, Handgelenk oder Finger; [17–19]) jeweils im Vergleich zu einer Operation bei insgesamt 320 Patientinnen eingesetzt wurden. Eine weitere Studie mit 52 Patient:innen untersuchte in 5 Einheiten die diakutane Fibrolyse, eine manuelle Technik zur Mobilisation und Behandlung von Schmerzen, im Vergleich zu einer Scheintherapie [26]. Eine Studie verwendete bei 22 Patient:innen eine Bindegewebsmassage [15] über einen Zeitraum von 6 Wochen, und eine weitere Studie überprüfte den Effekt verschiedener therapeutischer Massagetechniken [35] an 27 Patient:innen, ebenfalls über einen Zeitraum von 6 Wochen.

Zusammenfassend zeigten die Untersuchungen, die alle ein sehr niedriges Verzerrungspotenzial aufwiesen, dass manuelle Techniken bereits nach 5 Behandlungen klinisch relevante, positive Effekte bezogen auf die Reduktion der Schmerzintensität [15, 17, 19, 26, 52] und die Erhöhung der Funktionalität [15, 17, 19] bewirkten. Auch eine operative Dekompression des medianen Nervs [17–19] führte in Bezug auf die Schmerzreduktion zu klinisch relevanten Effekten [17, 19], jedoch erst nach einem Zeitraum von 3 Monaten [17] und 6 Monaten [18] zu einer Verbesserung der Funktionalität. Ein Jahr nach dem operativen Eingriff und der nachsorgenden physiotherapeutischen Behandlung befanden sich die Patient:innen auf einem annähernd gleichen Schmerz- und Funktionalitätsniveau.

Die instrumentell gestützte Bindegewebsmobilisation [15] zeigte in einer Studie mit einem sehr geringen Verzerrungspotenzial einen Vorteil in der Reduktion der Schmerzintensität gegenüber der klassischen Mobilisation des Bindegewebes. Die Bindegewebsmobilisation zeigte bereits nach 6‑wöchiger Behandlung eine klinisch relevante positive Wirkung, welche auch über einen Follow-upZeitraum bestehen blieb. In Bezug auf die Funktionalität erwiesen sich beide Mobilisationstechniken als klinisch relevant.

Nach gezielter Armmassage und genereller Nacken-Rücken-Massage [35] zeigten sich eine statistisch nichtsignifikante Reduktion der Symptomstärke sowie eine Verbesserung der Funktionalität der Hände. Die Ergebnisse sind allerdings in Bezug auf die mangelnde Transparenz in der Studienprotokollierung als wenig aussagekräftig zu werten und weisen keine klinische Relevanz auf.

### Physikalische Therapieformen (Ultraschall- und Lasertherapie)

Insgesamt 5 Studien analysierten den Effekt einer physikalischen Therapie in Form von Laser- und/oder Ultraschalltherapie im Vergleich zur Faszienmobilisation [42] und neurodynamischen Mobilisation [3, 8, 57, 58]. In den Untersuchungen mit insgesamt 300 Patient:innen über einen Zeitraum von 3 [42], 4 [3], 8 [8] und 10 Wochen [57, 58] ließ sich eine deutliche Überlegenheit der physiotherapeutischen Techniken gegenüber den physikalischen Techniken (Laser- und Ultraschall) mit einer klinisch relevanten Reduktion der Schmerzintensität [3, 8, 42, 57, 58] und Symptomstärke [42] sowie einer Verbesserung der Funktionalität der Hände [42, 57, 58] belegen. Daneben zeigte die physikalische Therapie ebenso klinisch relevante Verbesserungen in der Schmerzintensität [3, 8, 57, 58], es gibt jedoch keine eindeutigen Belege für eine Verbesserung der Funktionalität. Insgesamt wies nur eine Studie ein sehr geringes Verzerrungspotenzial auf [57], in dieser Studie wurden allerdings keine Unterscheidungen in den Postuntersuchungen zwischen den Gruppen durchgeführt. Eine Studie mit hochsignifikanten Unterschieden in den Postuntersuchungen [58] ist aufgrund mangelnder Transparenz mit einigen Unklarheiten in der Dokumentation als mäßig aussagekräftig zu werten.

### Yoga

Garfinkel et al. [20] verglichen bei 42 Patient:innen den Effekt einer yogabasierten Behandlung über einen 8‑wöchigen Zeitraum mit dem Effekt einer nächtlichen Schienung des Handgelenks. Im Anschluss an die Behandlung ließ sich für die yogabasierte Interventionsgruppe eine höhere, klinisch relevante Schmerzlinderung (VAS) im Vergleich zu einer Schienung feststellen. Jedoch ist aufgrund mangelnder Transparenz bezüglich fehlender Daten bei der statistischen Analyse den Ergebnissen nur eine mäßige Aussagekraft zuzuschreiben.

## Diskussion

Diese systematische Übersichtsarbeit zielte darauf ab, einen aktuellen Überblick über die Effektivität und klinische Relevanz physio- oder sporttherapeutischer Interventionen im Vergleich zu anderen Therapieformen oder Placebo/Warteliste anhand randomisierter, kontrollierter Studien zu geben. Eine Evidenz hinsichtlich der Effektivität der Behandlung des KTS wird bisher vor allem für operative Verfahren, Schienung oder medikamentöse Therapieformen beschrieben. Physio- bzw. sporttherapeutische Interventionen sind in der aktuellen Leitlinie der Arbeitsgemeinschaft der Wissenschaftlichen Medizinischen Fachgesellschaften (AWMF) nicht systematisch einbezogen worden.

Ergänzend zu früheren Übersichtsarbeiten [25, 32, 36–38, 41] fasst dieses Review den aktuellen Forschungsstand zu einzelnen nichtinvasiven oder nichtmedikamentösen Therapien auf der Basis von 26 Studien zusammen. Hierbei soll die Effektivität einzelner und kombinierter Physiotherapieformen abgebildet werden, um der klinischen Praxis gezielt Handlungsoptionen aufzuzeigen. Im Unterschied zu den bisherigen Übersichtsarbeiten schließt dieses Review alle Studien mit dem Schwerpunkt medikamentöser oder operativer Verfahren ohne physio- oder sporttherapeutischen Vergleich aus. Weiterhin erfolgte ein Ausschluss von Studien, die neben einer physio- und/oder sporttherapeutischen Intervention eine medikamentöse Behandlung oder eine weitere invasive Therapiemethode enthielten, was die aktuelle Arbeit von vorherigen Übersichtsarbeiten abgrenzt [25, 38, 41].

Manuelle Mobilisationstechniken bewirken bereits nach 2 Wochen relevante Verbesserungen

Als Ergebnis dieses Reviews kann festgehalten werden, dass manuelle Mobilisationstechniken bereits nach 2‑wöchiger Behandlung klinisch relevante Verbesserungen der Symptomstärke, der Schmerzintensität und der Funktionalität der Hände erzielten. Demgegenüber setzten positive Effekte durch eine operative Dekompression [17–19] erst zeitverzögert ein. Hinsichtlich der Verbesserung der Symptomstärke besonders effektiv scheinen Mobilisationstechniken für den N. medianus in Form der sogenannten Neurodynamik [47, 52, 55, 56] und der Faszienmanipulation zu sein [42].

Eine klinisch relevante Verringerung der Schmerzintensität zeigte sich vor allem durch neurodynamische Techniken [44, 51–54, 56], neurodynamische Techniken in Kombination mit Kinesiotaping [29], Karpalknochenmobilisation [44, 51, 53] sowie Gelenk- und Weichteilmobilisationen zur Verbesserung des „mechanical interface“ [52]. Dies beschreibt die Bereiche, in denen der N. medianus in enger anatomischer Beziehung zu seiner Umgebung steht, was insbesondere im Bereich des Handgelenks der Fall ist, aber auch an Ellbogen und Schultergelenk [15, 52]. Weiterhin wirksam zeigten sich manuelle Techniken [17, 19, 26], Faszienmanipulation [42], Neuromobilisation [3, 8, 57, 58] und Yoga [20]. Für die Verbesserung der Funktionalität erwiesen sich die Neuromobilisation [10, 52, 54, 56–59], Neuromobilisation in Kombination mit Kinesiotaping [2, 29, 59], Weichteilmobilisation [15], manuelle Therapie [17, 18] und Faszienmanipulation [42] als klinisch relevant.

Die neurodynamische Mobilisation zeigte sich in den Studien gegenüber der Karpalknochenmobilisation [44, 51, 53] in der Wirkgeschwindigkeit deutlich überlegen, aber vergleichbar mit der Wirkgeschwindigkeit der Weichteilmobilisation [52]. Ähnliches zeigte eine gezielte Massage gegenüber einer allgemeinen Massage mit einer leichten Überlegenheit in der Reduktion der Symptomstärke [35]. Physikalische Interventionen, wie Laser- oder Ultraschallbehandlungen, waren im Vergleich zu manuellen Interventionen deutlich weniger effektiv [3, 8, 42, 57, 58], zeigten aber in der Verbesserung der Schmerzintensität durchaus klinische Relevanz [3, 8, 57, 58]. Diese Erkenntnisse können einen wertvollen Ansatz zur Überarbeitung der zu aktualisierenden S3-Leitlinie [9] darstellen.

Darüber hinaus könnten physiotherapeutische Behandlungen auch effektiv mit weiteren nichtinvasiven Behandlungsoptionen kombiniert werden. Beispielsweise scheint eine Schienenversorgung in Kombination mit neurodynamischer Mobilisation effektiver zu sein als die alleinige physiotherapeutische Behandlung [10]. Ebenso kann der zusätzliche Einsatz von Kinesiotaping die Resultate der neurodynamischen Mobilisation verbessern [2, 29, 59].

In der bislang einzigen systematischen Studie zur konservativen Behandlung des KTS mit sporttherapeutischen Interventionen und mit subjektiver Bewertung durch die Patient:innen wurde Yoga [20] mit einer Standardbehandlung verglichen; es zeigte sich eine leichte Überlegenheit in der Schmerzlinderung.

Insgesamt zeigten alle sport- und physiotherapeutischen Interventionen den Vorteil, bereits nach einem Interventionszeitraum von 2 Wochen positive Effekte aufzuweisen sowie Operationsrisiken zu vermeiden und zu einer Ökonomisierung der Kosten beizutragen. Zudem eigneten sich einige der Übungen auch zur eigenständigen ambulanten Durchführung in Form eines Heimprogramms.

Zusammenfassend ist die Evidenz zu physio- und sporttherapeutischen Interventionen bezüglich der Schmerz- (18 Studien) und Symptomlinderung (16 Studien) sowie der Steigerung der Funktionalität der Hände (19 Studien) als moderat einzustufen. Aufgrund teilweise mangelnder Transparenz in der Studienprotokollierung sowie aufgrund eines möglichen schwerwiegenden Verzerrungspotenzials in einzelnen Fällen bedarf es weiterer qualitativ hochwertiger und präzise protokollierter Studien.

## Limitationen

Es wurden nur deutsch- und englischsprachige Publikationen eingeschlossen, die durch die Handsuche gefunden wurden oder in den Datenbanken PubMed, CINAHL oder Web of Science geführt werden. Zudem wurde der Fokus auf die subjektiven Bewertungen der behandelten Patient:innen in Form von Schmerzintensität und Funktionalität gelegt, was eine mögliche Limitierung in Bezug auf die Interpretation der Evidenz darstellen könnte. Eine Verbesserung objektiver Parameter wie der Nervenleitgeschwindigkeit kann nicht abgeleitet werden. Jedoch wird in neueren Studien die physiologische Messung, beispielsweise über die Nervenleitgeschwindigkeit als Goldstandard, insbesondere für leichte und mittelgradige periphere Kompressionsneuropathien kritisch diskutiert [47].

Eine Berücksichtigung kombinierter Interventionen von operativen und medikamentösen Therapieformen sowie der Schienung mit einer sport- und/oder physiotherapeutischen Therapie könnte weitere Aufschlüsse über die Effektivität dieser Behandlungsformen geben. In Anbetracht des Fokus der vorliegenden Übersichtsarbeit auf die Effekte rein sport- und/oder physiotherapeutischer Therapieformen ist diese kombinierte Behandlungsmethode für das Aufzeigen der Evidenz wenig aufschlussreich.

Abschließende Aussagen zu mittel- bis langfristigen Verbesserungen können aufgrund der vorhandenen Studien nur eingeschränkt getroffen werden, da nur wenige Studien langfristige Follow-up-Messungen berichteten.

## Fazit für die Praxis

Zusammenfassend bestehen für Patient:innen mit einem leichten bis milden Karpaltunnelsyndrom nichtoperative Therapiemöglichkeiten zur Reduktion der Schmerzen und zur Verbesserung der Funktionalität. Vor allem sind Erfolge durch physio- und sporttherapeutische Interventionen bereits nach 2 Wochen erzielbar und deren Ergebnisse sind langfristig (nach 12 Monaten) mit denen einer Operation vergleichbar bei gleichzeitiger Vermeidung von Operationsrisiken. Die daraus resultierende Evidenz für konservative Behandlungsmöglichkeiten, neben der etablierten Schienenversorung, sind auf Grundlage vorliegender Publikationen, mit zum Teil niedriger methodischer Qualität, als noch moderat einzustufen. Dennoch zeigen die ausgewählten Studien bereits Erfolge im Einsatz dieser Therapiemethoden.Daher sind weitere randomisierte, kontrollierte Studien hoher methodischer Qualität mit längeren Follow-up-Zeiträumen erforderlich, um eine klare Handlungsempfehlung formulieren zu können.

## Supplementary Information





## Abkürzungen

BCTQ FSS: Boston Carpal Tunnel Questionnaire, Functional Status Scale
BCTQ SSS: Boston Carpal Tunnel Questionnaire, Symptom Severity Scale
DN4: Pain Characteristics and Sensations Score
FBS: Functional Box Scale
KTS: Karpaltunnelsyndrom
NPRS: Numeric Pain Rating Scale
PRS: Pain Relief Scale
PSFS: Patient Specific Functional Scale
STP: Symptom Total Point Score
VAS: Visuelle Analogskala
