# Supplementary material for: Physiotherapy and sports therapeutic interventions for treatment of carpal tunnel syndrome: A systematic review
Source: Schmerz. 2022 Mar 14;36(4):256–65. [Article in German] doi: 10.1007/s00482-022-00637-x (PMC9300529; doi:10.1007/s00482-022-00637-x)
Supplement: Supplementary file 1 [file 482_2022_637_MOESM1_ESM.pdf]

Tabelle S1 Datenextraktionstabelle

Autor	Jahr	Journal	Intervention (IG)	Kontrolle (KG)	Diagnose	Patient:innen (IG)	Patient:innen (KG)	Alter (IG) (MW ± SD)	Alter (KG) (MW ± SD)	Dauer (IG)	follow-up	Mess-instrumente	Ergebnisse (IG) Score	Ergebnisse (KG) Score	P-Wert
Bardak et al. [10]	2009	Advances in therapy	Sehnen-Nerven-gleitübungen (Gruppe 3)<br><br>Sehnen-Nerven-gleitübungen und Standard-behandlung (Gruppe 2)	Standard-behandlung (Schiene & Injektionen) (Gruppe 1)	Lundborg classification & elektro-physiologische Untersuchung	35<br>(0 m)<br>(Gruppe 2)<br><br>35<br>(2 m)<br>(Gruppe 3)	41<br>(1 m)	26 ± 0,3<br>(Gruppe 2)<br><br>22 ± 9,9<br>(Gruppe 3)	33 ± 9,6	6 Wochen	nach 8 Wochen	Symptom total point score (STP)	Prä: 4,2 ± 0,8<br>Post:1,3 ± 1,6<br>(Gruppe 2)<br><br>Prä: 4,57 ± 0,95<br>Post: 4,11 ± 1,28<br>(Gruppe 3)	Prä:4,1 ± 1,1<br>Post:1,8 ± 1,9	Prä-Post:<br>IG Gr2:<br>< 0,001<br>IG Gr3:<br>= 0,007<br>KG:<br>< 0,001<br>KG vs. Gr 3<br>< 0,001<br>Gr 2 vs Gr3<br>< 0,001
												Functional status scale	Prä:15,5 ± 4,1<br>Post: 10,8 ± 4,2<br>(Gruppe 2)<br><br>Prä: 17,00 ± 7,19<br>Post: 15,20 ± 6,26<br>(Gruppe 3)	Prä: 14,8 ± 4<br>Post: 11 ± 3,4	Prä-Post:<br>IG Gr2:<br>< 0,001<br>IG Gr3:<br>= 0,001<br>KG:<br>< 0,001<br>KG vs. Gr 3<br>< 0,001<br>Gr 2 vs Gr3<br>< 0,001
Mohamed et al. [32]	2016	Egyptian Rheuma-tology and Reha-bilitation	Neuro-dynamische mobilisation	Standard-behandlung	Klinische & elektro-physiologische Untersuchung	18 w	10 w	34,05 ± 10,19		6 Wochen	Prä-Post	VAS	n.a.		Prä-post:<br>IG:<br>≤ 0,0001<br>KG:<br>≤ 0,0001<br>Zwischen Gruppen<br>= 0,003
												BCTQ (SSS)	n.a.		Prä-post:<br>IG:<br>≤ 0,0001<br>KG:<br>≤ 0,0001<br>Zwischen Gruppen<br>= 0,005
												BCTQ (FSS)	n.a.		Prä-post:<br>IG:<br>≤ 0,0001<br>KG:<br>≤ 0,0001<br>Zwischen Gruppen<br>n.s.

Autor	Jahr	Journal	Intervention (IG)	Kontrolle (KG)	Diagnose	Patient:innen (IG)	Patient:innen (KG)	Alter (IG) (MW ± SD)	Alter (KG) (MW ± SD)	Dauer (IG)	follow-up	Mess-instrumente	Ergebnisse (IG) Score	Ergebnisse (KG) Score	P-Wert
Sakr al. [43]	2019	Bioscience Research	Neuro-dynamische Mobilisation	Karpal-knochen-mobilisation	Klinische & elektro-physiologische Untersuchung	15 (w)	15 (w)	36,47 ± 6,58	36,40 ± 7,02	4 Wochen	Prä-Post	VAS	Prä:<br>7,80 ± 1,15<br><br>Post:<br>1,20 ± ,94	Prä:<br>7,93 ± ,88<br><br>Post:<br>1,07 ± ,96	Prä-Post<br>IG: < 0,001<br>KG:<br>< 0,001<br>Zwischen Gruppen<br>Post: n.s.
Schmid et al. [44]	2012	Journal of orthopaedic research	Heim-übungen: Sehnen- und Nerven-gleitübungen	Nächtliche Schienung	Klinische & elektro-physiologische Untersuchung	10	10	49,9 ± 12,5	57,9 ± 16,3	1 Woche	1 Woche	VAS<br><br>BCTQ (SSS)	Prä: 0,7 ± 1,3<br>Post: 0,8 ± 1,4<br><br>Prä: 1,8 ± 0,5<br>Post: 1,5 ± 0,6	Prä: 1,2 ± 2,1<br>Post: 1,1 ± 1,1<br><br>Prä: 2,0 ± 0,4<br>Post: 1,7 ± 0,4	n.s.<br><br>Prä-Post:<br>IG: < 0,004<br>KG:<br><0,004)
Shem et al. [46]	2020	Journal of Hand Therapy	Stretching	Schein-therapie	Klinische & elektro-physiologische Untersuchung	19 (32 Hände)	17 (32 Hände)	50,05 ± 9,71	48,18 ± 7,18	4x/Tag<br>6 Wochen	Prä-Post	BCTQ (SSS)<br><br>BCTQ (FSS)	Prä:<br>30,8 ± 8,76<br><br>Post:<br>27,0 ± 10,0<br><br>Prä:<br>29,3 ± 9,08<br><br>Post:<br>26,9 ± 11,3	Prä:<br>32,3 ± 7,68<br><br>Post:<br>29,3 ± 7,19<br><br>Prä:<br>23,3 ± 10,3<br><br>Post:<br>23,1 ± 9,57	IG: 0,007<br>KG: n.s.<br><br>IG: n.s.<br>KG: n.s.
Tal-Akabi et al. [48]	2000	Manual therapy	Neuro-dynamische mobilisation (Gruppe 1)<br><br>Karpal-knochen-mobilisation (Gruppe 2)	Keine Behandlung	Klinische & elektro-physiologische Untersuchung	7 (Gruppe 1)<br><br>7 (Gruppe 2)	7	47,1 ± 14,8 (range 29-85)<br><br>m:w = 1:2	3 Wochen	3 Wochen	Prä-Post	VAS<br><br>Pain relief scale (PRS)<br><br>Functional box scale (FBS)	Gruppe 1<br>Prä: 2,42<br>Post: 1,57<br><br>Gruppe 2<br>Prä: 2,29<br>Post: 0,71<br><br>Gruppe 1<br>Post: 3,14<br><br>Gruppe 2<br>Post: 3,71<br><br>Gruppe 1<br>Prä: 2<br>Post: 1,14<br><br>Gruppe 2<br>Prä: 2<br>Post: 0,71	Prä: 2<br>Post: 2,14<br><br>Post: 0<br><br>Prä: 2,42<br>Post: 2,42	Prä-post:<br>IG 1: < 0,02<br>IG 2:<br>< 0,001<br>KG: n.s.<br><br>1 vs. 2 vs. 3: < 0,05<br><br>n.s.

Autor	Jahr	Journal	Intervention (IG)	Kontrolle (KG)	Diagnose	Patient:innen (IG)	Patient:innen (KG)	Alter (IG) (MW ± SD)	Alter (KG) (MW ± SD)	Dauer (IG)	follow-up	Mess-instrumente	Ergebnisse (IG) Score	Ergebnisse (KG) Score	P-Wert
Talebi et al., [49]	2020	Caspian Journal of Internal Medicine	Weichteil-mobilisation	Neuro-dynamische Mobilisation	Klinische & elektro-physiologische Untersuchung	15	15	48,86 ± 8,94	51,46 ± 9,62	3x/4 Wochen	Prä-post	VAS	Prä: 6,80 ± 1,65<br>Post: 3,93 ± 1,90	Prä: 6,40 ± 1,45<br>Post: 3,53 ± 2,23	Prä-post<br>IG: < 0,001<br>KG:<br>< 0,001<br>Zwischen Gruppen<br>n.s.
												BCTQ (SSS)	Prä: 30,13 ± 8,95<br>Post: 21,73 ± 8,22	Prä: 30,66 ± 7,82<br>Post: 19,26 ± 5,48	Prä-post<br>IG: < 0,001<br>KG:<br>< 0,001<br>Zwischen Gruppen<br>n.s.
												BCTQ (FSS)	Prä: 19,33 ± 8,05<br>Post: 14,53 ± 5,13	Prä: 17,20 ± 6,77<br>Post: 12,33 ± 5,48	Prä-post<br>IG: = 0,001<br>KG:<br>= 0,001<br>Zwischen Gruppen<br>n.s.
Vikranth et al. [50]	2015	International Journal of Physio-therapy	Karpal-knochen Mobilisation	Neuro-dynamische Mobilisation	positiver Phalen Test, positiver Tinels Test,<br>positiver ULTT2,<br>VAS ≥ 4	15 (5 m)	15 (8 m)	40,73 ± 9,86	40,33 ± 9,41	2 Wochen	Prä-Post	VAS	Prä:<br>5,53 ± 1,25<br>Post:<br>4,07 ± 1,39	Prä:<br>5,60 ± 1,40<br>Post:<br>3,67 ± 1,23	Prä-Post:<br>IG: < 0,001<br>KG: < 0,001<br>Zwischen Gruppen<br>Post: n.s.
												BCTQ (SSS)	Prä:<br>2,48 ± 0,56<br>Post:<br>2,07 ± 0,56	Prä:<br>2,56 ± 0,62<br>Post:<br>2,03 ± 0,46	Prä-Post:<br>IG: < 0,001<br>KG: < 0,001<br>Zwischen Gruppen<br>Post: n.s.
												BCTQ (FSS)	Prä:<br>2,32 ± 0,79<br>Post:<br>1,96 ± 0,70	Prä:<br>2,56 ± 0,78<br>Post:<br>2,05 ± 0,69	Prä-Post:<br>IG: < 0,001<br>KG: < 0,001<br>Zwischen Gruppen<br>Post: n.s.

Autor	Jahr	Journal	Intervention (IG)	Kontrolle (KG)	Diagnose	Patient:innen (IG)	Patient:innen (KG)	Alter (IG) (MW ± SD)	Alter (KG) (MW ± SD)	Dauer (IG)	follow-up	Mess-instrumente	Ergebnisse (IG) Score	Ergebnisse (KG) Score	P-Wert
Wolny et al. [51]	2018a	Journal of Manipulative and Physio-logical Thera-peutics	Neuro-dynamische Techniken	Keine Behandlung	Klinische Untersuchung	102 (12 m)	87 (8 m)	52,6 ± 9,3	53,1 ± 8,9	10 Wochen	Prä-Post	BCTQ (SSS)<br><br>BCTQ (FSS)	Prä: 5,5<br>Post: 3,03<br>Prä: -<br>Post: 2,88	Prä: 5,6<br>Post: 3,04<br>Prä: -<br>Post: 2,91	n.s.<br>n.s.<br>-<br>n.s.
Wolny et al. [52]	2018b	Archives of physical medicine and rehabilitation	Neuro-dynamische Techniken	Schein-therapie	Klinische & elektro-physiologische Untersuchung	78 (8 m)	72 (7 m)	54,2 ± 9,48	52,2 ± 10,4	10 Wochen	Prä-Post	NPRS<br><br>BCTQ (SSS)<br><br>BCTQ (FSS)	Prä: 5,95 ± 1,54<br>Post: 1,42 ± 1,02<br><br>Prä: 2,99 ± 0,67<br>Post: 1,77 ± 0,48<br><br>Prä: 2,84 ± 0,72<br>Post: 1,94 ± 0,61	Prä: 5,58 ± 1,27<br>Post: 5,42 ± 0,99<br><br>Prä: 2,88 ± 0,72<br>Post: 2,86 ± 0,72<br><br>Prä: 3,04 ± 0,64<br>Post: 3,09 ± 0,68	IG: p < 0,01<br>KG: n.s.<br>Zwischen Gruppen Post:<br>p < 0,01<br><br>IG: p < 0,01<br>KG: n.s.<br>Zwischen Gruppen Post:<br>p < 0,01<br><br>IG: p < 0,01<br>KG: n.s.<br>Zwischen Gruppen Post:<br>p < 0,01
Wolny et al. [53]	2019	Clinical Reha-bilitation	Neuro-dynamische Techniken	Keine Behandlung	Elektro-physiologische Untersuchung	58 (6 m)	45 (5 m)	54,6 ± 9,7	53,1 ± 10,1	10 Wochen /2x Woche	Prä-Post	NPRS<br><br>BCTQ (SSS)<br><br>BCTQ (FSS)	Prä: 5,86 ± 1,46<br>Post: 1,38 ± 1,01<br>Prä: 3,03 ± 0,65<br>Post: 1,08 ± 0,68<br>Prä: 2,82 ± 0,71<br>Post: 1,96 ± 0,64	Prä: 5,71 ± 1,34<br>Post: 5,46 ± 1,05<br>Prä: 2,92 ± 0,71<br>Post: 2,87 ± 0,68<br>Prä: 2,99 ± 0,67<br>Post: 2,87 ± 0,71	n.s.<br><0,01<br>n.s.<br><0,01<br>n.s.<br><0,01
**Nerven-Sehnengleitübungen und Kinesiotaping**															
Aktürk et al. [2]	2018	International League of Associations for Rheumatolo gy	Kinesiotape & Nerven-Sehen-gleitübungen	Schienung & Nerven-Sehen-gleitübungen	Elektro-myographie	28 Hände (22 w)	30 Hände (29 w)	49,2 ± 11,7	48,2 ± 9,2	10 Einheiten (5 Wochen)	Prä-Post	BCTQ (SSS)<br><br>BCTQ (FSS)	Prä: 33,1 ± 8,79<br>Post: 22,7 ± 8,01<br>Prä: 21,2 ± 9,17<br>Post: 12,14 ± 4,9	Prä: 33,0 ± 4,23<br>Post: 32,7 ± 4,21<br>Prä: 19,4 ± 7,67<br>Post: 18,9 ± 7,46	n.s.<br>0,001<br>n.s.<br>0,01

Autor	Jahr	Journal	Intervention (IG)	Kontrolle (KG)	Diagnose	Patient:innen (IG)	Patient:innen (KG)	Alter (IG) (MW ± SD)	Alter (KG) (MW ± SD)	Dauer (IG)	follow-up	Mess-instrumente	Ergebnisse (IG) Score	Ergebnisse (KG) Score	P-Wert
Külcü et al. [28]	2016	Turkish Journal of medical Sciences	1:<br>Kinesiotape & Heim-übungen, Sehnen-gleitübungen	2: Plazebo Kinesiotape & Heim-übungen, Sehnen-gleitübungen<br><br>3: Orthese & Heim-übungen, Sehnen-gleitübungen	Klinische Untersuchung, Symptome < 1 Jahr, sensorische Untersuchung	1:<br>13 (20 Hände) (1 m)	2:<br>13 (20 Hände) (0 m)<br><br>3:<br>14 (20 Hände) (1 m)	1:<br>49,8 ± 11,5	2:<br>48,95 ± 6,0<br><br>3:<br>51,3 ± 8,3	4 Wochen	Prä-Post	VAS	Prä:<br>1: 6,6 ± 2,1<br><br>Post:<br>1: 4,1 ± 2,7	Prä:<br>2: 5,8 ± 3,2<br>3: 6,1 ± 2,9<br><br>Post:<br>2: 3,9 ± 2,8<br>3: 5,7 ± 3,1	1: = 0,001<br>2: = 0,009<br>3: = 0,030<br><br>Zwischen Gruppen n.s.
												pain characteristics and sensations (DN4)	Prä:<br>1: 5,5 ± 2,3<br><br>Post:<br>1: 3,7 ± 2,3	Prä:<br>2: 4,6 ± 2,6<br>3: 4,5 ± 1,6<br><br>Post:<br>2: 3,0 ± 2,4<br>3: 3,7 ± 2,0	1: = 0,005<br>2: = 0,003<br>3: = 0,024<br><br>Zwischen Gruppen n.s.
												BCTQ (SSS)	Prä:<br>1: 32 ± 8,4<br><br>Post:<br>1: 20 ± 7,5	Prä:<br>2: 33 ± 10,7<br>3: 31,6 ± 8,4<br><br>Post:<br>2: 24,4 ± 8,0<br>3: 28,7 ± 11,8	1: <0,0001<br>2: <0,0001<br>3: = 0,036<br><br>Zwischen Gruppen = 0,024 (1/3)
												BCTQ (FSS)	Prä:<br>1: 23,1 ± 6,0<br><br>Post:<br>1: 16,2 ± 5,4	Prä:<br>2: 19,7 ± 8,4<br>3: 21,7 ± 7,0<br><br>Post:<br>2: 16,3 ± 5,8<br>3: 19,7 ± 19,7	1: = 0,001<br>2: n.s.<br>3: n.s.<br><br>Zwischen Gruppen = 0,017 (1/3)
Yildirim et al. [56]	2018	Turkish Journal of Medical Sciences	Kinesiotape & Nerven-Sehnen-gleitübungen	Nerven-Sehen-gleitübungen	Klinische & elektro-physiologische Untersuchung	11 (0 m)<br>19 Hände	10 (1 m)<br>19 Hände	48,81 ± 6,40	48,70 ± 7,61	6 Wochen	Prä<br>3 Wochen<br>6 Wochen	BCTQ (SSS)	Prä:<br>33,26 ± 8,40<br><br>3 Wochen:<br>19,84 ± 4,65<br><br>6 Wochen:<br>22,15 ± 7,45	Prä:<br>31,60 ± 9,48<br><br>3 Wochen:<br>25,66 ± 8,79<br><br>6 Wochen:<br>19,06 ± 7,18	Zwischen Gruppen:<br><br>= 0,02<br><br>n.s.
												BCTQ (FSS)	Prä:<br>20,57 ± 6,58<br><br>3 Wochen:<br>10,89 ± 4,40<br><br>6 Wochen:<br>13,26 ± 5,52	Prä:<br>17,40 ± 5,55<br><br>3 Wochen:<br>13,73 ± 4,13<br><br>6 Wochen:<br>11,26 ± 4,63	 <br>= 0,01<br><br>n.s.
**Mobilisation (Weichteilmobilisation, Mobilisation assoziierter anatomischer Bereiche, Bindegewebsmobilisation, neurodynamische Mobilisation, Karpalknochenmobilisation, Faszienmobilisation, allgemeine Massage)**															

Autor	Jahr	Journal	Intervention (IG)	Kontrolle (KG)	Diagnose	Patient:innen (IG)	Patient:innen (KG)	Alter (IG) (MW ± SD)	Alter (KG) (MW ± SD)	Dauer (IG)	follow-up	Mess-instrumente	Ergebnisse (IG) Score	Ergebnisse (KG) Score	P-Wert
Burke et al. [15]	2007	Journal of manipulative and physiological therapeutics	Graston-Instrument gestützte Weichteil-mobilisation	Weichteil-mobilisation	Klinische & elektro-physiologische Untersuchung	12 (2 m)	10 (1 m)	39,8 ± 8,8	43,4 ± 5,3	6 Wochen	Direkt nach Inter-vention & 3 Monate danach	VAS	Prä: 6,15 ± 2,66	Prä: 6,05 ± 1,79	
													Immed: 0,98 ± 1,25	Immed: 1,54 ± 1,96	-
													3 Monate 0,92 ± 1,10	3 Monate 3,37 ± 2,88	Zwischen Gruppen < 0,05
												BCTQ (SSS)	Prä: 3,0 ± 0,73	Prä: 2,7 ± 0,64	
													Immed: 1,8 ± 0,74	Immed: 1,9 ± 0,39	n.s.
													3 Monate 1,8 ± 0,61	3 Monate 2,2 ± 0,59	n.s.
												BCTQ (FSS)	Prä: 2,2 ± 0,88	Prä: 2,4 ± 0,85	
													Immed: 1,6 ± 0,65	Immed: 1,7 ± 0,70	n.s.
													3 Monate 1,6 ± 0,72	3 Monate 1,7 ± 0,68	n.s.
Fernández-de-Las Penas et al. [17]	2015	The Journal of pain	Manuelle Therapie	Operation	Klinische & elektro-physiologische Untersuchung	60 (0 m)	60 (0 m)	47 ± 10	46 ± 9	3 Wochen	1, 3, 6, 12 Monate	NPRS (Durchschnitt)	Prä: 4,8 ± 1,5	Prä: 4,9 ± 2,2	Zwischen Gruppen <0,001
													1 Monat 1,4 ± 1,9	1 Monat 3,4 ± 2,3	
													3 Monate 1,1 ± 1,8	3 Monate 2,5 ± 2,1	<0,001
													6 Monate 1,1 ± 1,6	6 Monate 1,8 ± 2,5	n.s.
													12 Monate 1,2 ± 1,8	12 Monate 1,3 ± 1,9	n.s.
												BCTQ (SSS)	Prä: 2,5 ± 0,7	Prä: 2,7 ± 0,6	
													1 Monat 1,6 ± 0,5	1 Monat 1,7 ± 0,5	n.s.
													3 Monate 1,6 ± 0,6	3 Monate 1,6 ± 0,4	n.s.
													6 Monate 1,6 ± 0,6	6 Monate 1,5 ± 0,5	n.s.
													12 Monate 1,5 ± 0,5	12 Monate 1,5 ± 0,5	n.s.
												BCTQ (FSS)	Prä: 2,3 ± 0,5	Prä: 2,4 ± 0,6	
													1 Monat 1,5 ± 0,4	1 Monat 2,3 ± 0,7	<0,001
													3 Monate 1,5 ± 0,5	3 Monate 1,8 ± 0,7	<0,001

Autor	Jahr	Journal	Intervention (IG)	Kontrolle (KG)	Diagnose	Patient:innen (IG)	Patient:innen (KG)	Alter (IG) (MW ± SD)	Alter (KG) (MW ± SD)	Dauer (IG)	follow-up	Mess-instrumente	Ergebnisse (IG) Score	Ergebnisse (KG) Score	P-Wert
													6 Monate<br>1,5 ± 0,5<br><br>12 Monate<br>1,5 ± 0,5	6 Monate<br>1,6 ± 0,6<br><br>12 Monate<br>1,5 ± 0,6	n.s.<br><br>n.s.
Fernandez-de-Las-Peñas et al. [18]	2017a	The Journal of orthopaedic and sports physical therapy	Manuelle Therapie	Operation	Klinische & elektro-phyiologische Untersuchung	50<br>(0 m)	50<br>(0 m)	46 ± 9	47 ± 8	3 Wochen	1, 3, 6, 12 Monate	BCTQ (SSS)	Prä: 2,5 ± 0,7<br><br>1 Monat<br>1,7 ± 0,6<br><br>3 Monate<br>1,7 ± 0,7<br><br>6 Monate<br>1,6 ± 0,6<br><br>12 Monate<br>1,6 ± 0,6	Prä: 2,6 ± 0,6<br><br>1 Monat<br>1,7 ± 0,5<br><br>3 Monate<br>1,5 ± 0,5<br><br>6 Monate<br>1,4 ± 0,5<br><br>12 Monate<br>1,4 ± 0,5	 <br>n.s.<br><br>n.s.<br><br>n.s.<br><br>n.s.
												BCTQ (FSS)	Prä: 2,1 ± 0,5<br><br>1 Monat<br>1,6 ± 0,6<br><br>3 Monate<br>1,6 ± 0,7<br><br>6 Monate<br>1,6 ± 0,5<br><br>12 Monate<br>1,6 ± 0,6	Prä: 2,2 ± 0,5<br><br>1 Monat<br>2,3 ± 0,7<br><br>3 Monate<br>1,7 ± 0,7<br><br>6 Monate<br>1,6 ± 0,6<br><br>12 Monate<br>1,5 ± 0,6	 <br><0,001<br><br>n.s.<br><br>n.s.<br><br>n.s.
Fernández-de-las-Peñas et al. [19]	2017b	European Journal of Pain	Manuelle Therapie (Weichteil-mobilisation & Sehnen-Nerven-gleitübungen)	Operation	Klinische & elektro-physiologische Untersuchung	50<br>(0 m)	50<br>(0 m)	47 ± 10	48 ± 9	3 Wochen & Heim-übungen 2x/Tag über einen Monat	3, 6, 9, 12 Monate	NPRS	Grafik ca.<br>Prä: 4,2<br><br>3 Monate<br>1,75<br><br>6 Monate<br>1,75<br><br>9 Monate<br>1,75<br><br>12 Monate<br>1,5	Grafik ca.<br>Prä: 4,4<br><br>3 Monate<br>3,2<br><br>6 Monate<br>2,3<br><br>9 Monate<br>2,0<br><br>12 Monate<br>1,5	 <br><0,001<br><br>n.s.<br><br>n.s.<br><br>n.s.
Jiménez del Barrio et al. [26]	2018	Clinical Reha-bilitation	Manuelle Therapie (diakutane Fibrolyse)	Schein-therapie	Klinische Untersuchung	25 (36 Hände)<br>(6 m)	27 (36 Hände)<br>(5 m)	44,97 ± 9,34	48,83 ± 7,98	5 x je 20 min (Ø 17,77 Tage)	Prä-Post &<br>1 Monat follow-up	VAS	Prä: 3,32 ± 2,1<br><br>Post: 0,42 ± 0,82<br><br>1 Monat:<br>0,93 ± 1,78	Prä:3,6 ± 2,29<br><br>Post: 3,48 ± 2,67<br><br>1 Monat:<br>3,99 ± 2,42	Zwischen Gruppen<br><br>Post < 0,01<br><br>follow-up<br>< 0,01

Autor	Jahr	Journal	Intervention (IG)	Kontrolle (KG)	Diagnose	Patient:innen (IG)	Patient:innen (KG)	Alter (IG) (MW ± SD)	Alter (KG) (MW ± SD)	Dauer (IG)	follow-up	Mess-instrumente	Ergebnisse (IG) Score	Ergebnisse (KG) Score	P-Wert
Moraska et al. [34]	2008	Journal of alternative and com-plementary medicine	Gezielte Massage (Arm-massage KTS) (TM)	Generelle Massage (Nacken-Rücken-massage) (GM)	Klinische Untersuchung	14	13	47,0 ± 8,80	50,3 ± 15,1	6 Wochen	Nach 11 Einheiten und 4 Wochen nach der Be-handlung	BCTQ (SSS)<br><br>BCTQ (FSS)	(Grafik) Prä: 2,2<br>Post: 1,8 ± 0,2<br><br>(Grafik) Prä: 1,75<br>Post: 1,75	(Grafik) Prä: 2,3<br>Post: 1,9 ± 0,2<br><br>(Grafik) Prä: 2,0<br>Post: 1,7	n.s.<br><br>n.s.
**Physikalische Therapieformen (Laser- und Ultraschalltherapie)**															
**Autor**	**Jahr**	**Journal**	**Intervention (IG)**	**Kontrolle (KG)**	**Diagnose**	**Patient:innen (IG)**	**Patient:innen (KG)**	**Alter (IG) (MW ± SD)**	**Alter (KG) (MW ± SD)**	**Dauer (IG)**	**follow-up**	**Mess-instrumente**	**Ergebnisse (IG)**	**Ergebnisse (KG)**	**P-Wert**
Alam et al. [3]	2018	Biomedical Research and Therapy	Neuro-dynamische Mobilisation	Physi-kalische Therapie (Ultraschall)	Elektro-diagnostische Tests, Phalen Test	24 (12m/12w)	24 (2m/22w)	m: 42,08 ± 7,87<br>w: 40,8 ± 8,02	m: 37,50 ± 17,68<br>w: 40,91 ± 9,69	12 Einheiten (4 Wochen)	Prä-Post	VAS	Prä: 6,41 ± 1,21<br>Post: 0,70 ± 0,69	Prä: 7,37 ± 0,71<br>Post: 4,41 ± 1,05	Prä-Post<br>IG: < 0,01<br>KG: < 0,01<br>Zwischen Gruppen Post:<br>n.s.
Atya et al. [8]	2011	Life Science Journal	Nerven-Sehnen-gleitübungen	Physi-kalische Therapie (Laser-therapie)	Klinische & elektro-physiologische Untersuchung	15 w	15 w	38,07 ± 2,25	38,47 ± 2,29	3x/Tag<br>10 x in 2 Monaten	Prä-Post	VAS	Prä: 7,53 ± 1,5<br>Post: 5,2 ± 1,52	Prä: 7,13 ± 1,3<br>Post: 2,86 ± 1,30	Prä-Post<br>IG: < 0,001<br>KG: < 0,001<br>Zwischen Gruppen Post:<br>< 0,05
Pratelli et al. [41]	2015	Journal of bodywork and movement therapies	Faszien-manipulation	Laser-therapie	Klinische & elektro-physiologische Untersuchung	35 sympt. Hände	35 sympt. Hände	54,2 (range 38-74)<br>29 w, 13 m	3 Wochen (IG)<br><br>5 Tage (KG)	10 Tage nach letzter Behandlung<br><br>3 Monate	Prä-Post	VAS<br><br><br><br><br>BCTQ (SSS)<br><br><br><br><br>BCTQ (FSS)	Prä: 6,00 ± 2,6<br><br>Post 10 Tage:<br>0,8 ± 0,96<br><br>3 Monate<br>0,71 ± 0,93<br><br>Prä: 3,03 ± 0,77<br><br>Post 10 Tage:<br>1,36 ± 0,27<br><br>3 Monate<br>1,28 ± 0,28<br><br>Prä: 3,1 ± 0,98<br><br>Post 10 Tage:1,41 ± 0,3<br><br>3 Monate<br>1,32 ± 0,32	Prä: 5,51 ± 2,24<br><br>Post 10 Tage:<br>5 ± 2,07<br><br>3 Monate<br>5,03 ± 2,02<br><br>Prä: 3,05 ± 0,35<br><br>Post 10 Tage:<br>2,67 ± 0,47<br><br>3 Monate<br>3,00 ± 0,31<br><br>Prä: 2,9 ± 0,89<br><br>Post 10 Tage:<br>2,58 ± 0,79<br><br>3 Monate<br>2,63 ± 0,94	<0,0001<br><br><0,0001<br><br><0,0001<br><br><0,0001<br><br><0,0001<br><br><0,0001
Wolny et al. [55]	2014	Phy-sikalische Medizin	Neuro-mobilisation (manuelle Techniken &	Physi-kalische Therapie (Ultraschall-	Klinische Untersuchung	20 (1 m)	20 (3 m)	53,4 ± 10,73	53,00 ± 9,41	20 Einheiten/2x Woche<br>10 Wochen	Prä-Post	NPRS	Prä:<br>6,21 ± 1,83<br><br>Post:<br>1,5 ± 1,29	Prä:<br>5,73 ± 1,56<br><br>Post:<br>4,5 ± 1,81	Prä-Post<br>IG: < 0,001<br>KG:<br>< 0,001

Autor	Jahr	Journal	Intervention (IG)	Kontrolle (KG)	Diagnose	Patient:innen (IG)	Patient:innen (KG)	Alter (IG) (MW ± SD)	Alter (KG) (MW ± SD)	Dauer (IG)	follow-up	Mess-instrumente	Ergebnisse (IG) Score	Ergebnisse (KG) Score	P-Wert
			Neuromobilisation)	& Laser-therapie)								BCTQ (SSS)<br><br>BCTQ (FSS)	Prä:<br>2,90 ± 0,75<br>Post:<br>1,64 ± 0,47<br><br>Prä:<br>2,79 ± 0,75<br>Post:<br>1,85 ± 0,49	Prä:<br>3,10 ± 0,65<br>Post:<br>2,79 ± 0,77<br><br>Prä:<br>3,17 ± 0,69<br>Post:<br>3,01 ± 0,68	Zwischen Gruppen Post: <0,001<br>Prä-Post IG: < 0,001 KG: n.s. Zwischen Gruppen Post: < 0,001<br>Prä-Post IG: < 0,001 KG: n.s. Zwischen Gruppen Post: <0,001
Wolny et al. [54]	2017	Journal of Manipulative and Physiological Therapeutics	Manuelle Therapie (neurodynamische Technik, funktionelle Massage Trapezius & Handgelenk-mobilisierung)	Physikalische Therapie (Laser-therapie)	Klinische & elektro-physiologische Untersuchung	70	70	53,1 ± 8,7	51,5 ± 10,3	10 Wochen	1 Monat	NPRS<br>BCTQ (SSS)<br>BCTQ (FSS)	Prä: 5,72 ± 1,49<br>Post: 1,47 ± 1,20<br>Prä:2,97 ± 0,63<br>Post: 1,78 ± 0,47<br>Prä: 2,80 ± 0,94<br>Post: 1,90 ± 0,62	Prä: 5,25 ± 1,75<br>Post: 3,58 ± 1,93<br>Prä: 2,94 ± 0,74<br>Post: 2,57 ± 0,77<br>Prä: 2,77 ± 0,94<br>Post: 2,55 ± 0,95	<0,01<br><0,01<br><0,01

Yoga

Autor	Jahr	Journal	Intervention (IG)	Kontrolle (KG)	Diagnose	Patient:innen (IG)	Patient:innen (KG)	Alter (IG) (MW ± SD)	Alter (KG) (MW ± SD)	Dauer (IG)	follow-up	Mess-instrumente	Ergebnisse (IG)	Ergebnisse (KG)	P-Wert
Garfinkel et al. [20]	1998	JAMA	Yoga	Schiene	Klinische & elektro-physiologische Untersuchung	22	20	48,9 (range 17-68)	48,7 (range 18-70)	8 Wochen	8 Wochen	Schmerz letzte Woche (VAS)	Prä: 5,0 ± 2,8<br>Post: 2,9 ± 2,2	Prä: 5,2 ± 2,1<br>Post: 4,3 ± 2,2	IG: = 0,02<br>KG: n.s.
